# Traditional Fermented Foods and Beverages from around the World and Their Health Benefits

**DOI:** 10.3390/microorganisms10061151

**Published:** 2022-06-02

**Authors:** Leonel Cuamatzin-García, Paola Rodríguez-Rugarcía, Elie Girgis El-Kassis, Georgina Galicia, María de Lourdes Meza-Jiménez, Ma. del Rocío Baños-Lara, Diego Salatiel Zaragoza-Maldonado, Beatriz Pérez-Armendáriz

**Affiliations:** 1 Biological Science Department, Universidad Popular Autónoma del Estado de Puebla, Puebla 72410, Mexico; leocuamatzin@gmail.com (L.C.-G.); paola.rdz.rugarcia@gmail.com (P.R.-R.); eliegirgis.elkassis@upaep.mx (E.G.E.-K.); 2 Centre for Genomics and Oncological Research (GENYO), Pfizer-University of Granada-Junta de Andalucía, 18016 Granada, Spain; georgina.galicia@gmail.com; 3 Department of Health Sciencies, School of Nutrition, Universidad Popular Autónoma del Estado de Puebla, Puebla 72410, Mexico; marialourdes.meza@upaep.mx; 4 Oncological Research Center, Una Nueva Esperanza, Universidad Popular Autónoma del Estado de Puebla, Puebla 72410, Mexico; marocio.banos@upaep.mx (M.d.R.B.-L.); diegozaragoza@unanuevaesperanza.mx (D.S.Z.-M.); 5 School of Medicine, Universidad Popular Autónoma del Estado de Puebla, Puebla 72410, Mexico

**Keywords:** traditional fermented foods and beverages, functional food, gut microbiota, prebiotics, probiotics

## Abstract

Traditional fermented foods and beverages play an important role in a range of human diets, and several experimental studies have shown their potential positive effects on human health. Studies from different continents have revealed strong associations between the microorganisms present in certain fermented foods (e.g., agave fructans, kefir, yeats, kombucha, chungkookjang, cheeses and vegetables, among others) and weight maintenance, reductions in the risk of cardiovascular disease, antidiabetic and constipation benefits, improvement of glucose and lipids levels, stimulation of the immunological system, anticarcinogenic effects and, most importantly, reduced mortality. Accordingly, the aim of this review is to corroborate information reported in experimental studies that comprised interventions involving the consumption of traditional fermented foods or beverages and their association with human health. This work focuses on studies that used fermented food from 2014 to the present. In conclusion, traditional fermented foods or beverages could be important in the promotion of human health. Further studies are needed to understand the mechanisms involved in inflammatory, immune, chronic and gastrointestinal diseases and the roles of fermented traditional foods and beverages in terms of preventing or managing those diseases.

## 1. Introduction

Traditional fermented foods and beverages (TFFB) occupy an important place in human diets. The earliest evidence of the use of fermented foods and beverages comes from Asia from around 8000 B.C in the form of vessels found in archeological areas [1]. Nowadays, fermented foods and beverages are defined as “foods or beverages produced through controlled microbial growth with conversion of food components through enzymatic action” [2].

Different fermented foods are consumed around the world. It has been reported that between 5% to 40% of all food consumed by humans belongs to this group [3]. Importantly, the positive effects of fermented foods on health have made them essential in human diets.

Fermented foods and beverages work in the human body through the presence of functional microorganisms and their ability to transform the chemical elements of raw materials of plants and animals. During food fermentation, the bio-availability of nutrients increases, stimulating probiotic and prebiotic functions, thereby improving the nutritional properties and health benefits of the food in question [4]. To achieve this effect, the bacteria must remain fictile during gastric transit in order to reach the site of action. Microorganisms have to reach the intestinal tract alive to perform their functions, including modifications of the gut microbiota and fermentation, among others [5].

Throughout history, humans have found different ways to satisfy their hunger. Different traditional fermented foods, such as yogurt, cheeses, crème fraiche, fermented sausages, sourdough bread, soy sauce, fish sauce, fermented vegetables, including “miso” (fermented soybeans), “kimchi” (fermented spicy cabbage), “sauerkraut” (fermented cabbage), and “surströmming” (fermented herring) or different beverages like “kefir” (fermented milk), “kombucha” (fermented tea), beer, wine or “kvass”, exist around the world [6,7,8,9]. These are some examples of the most popular fermented foods and beverages; however, people back then did not know the benefits of TFFB.

Even though there is no legislative definition of functional foods, Konstantinidis et al., [10] described them as “foods that possess constructive effects on target functions into the human organism, beyond nutritional effects, aiming health promotion and wellbeing and/or the reduction of chronic diseases” [10]. Therefore, fermented foods and beverages are not only considered to provide nutrients to the body; benefits have been suggested regarding certain diseases such as diabetes, obesity, metabolic syndrome, heart disorders, inflammatory diseases, bacterial and viral infections, thrombosis, allergy and diarrhea. Additionally, they exert vasodilatory actions and provide beneficial effects against certain types of cancer, as well as gastrointestinal disorders, among others [11,12,13,14,15,16].

**Figure 1:**
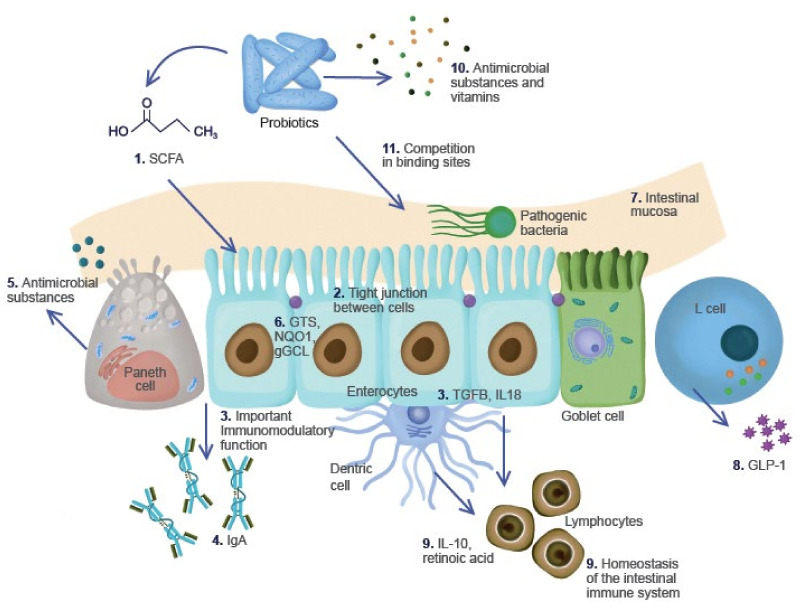
Probiotic functions. (1) Short-chain fatty acids (SCFA: butyrate, propionate, acetate) are the main sources of energy of intestinal cells. (2) The adequate function of enterocytes increases the barrier function and prevents the passage of pathogens. (3) Enterocytes inhibit the production of pro-inflammatory cytokines IL-6*, TNF-α* and IFN-γ*, and stimulate the production of TGFB and IL-8* for lymphocyte recruitment, which maintains the immune balance. (4) With the help of Paneth cells, they also produce immunoglobulin A (IgA). (5) Paneth cells produce antimicrobial substances (alpha defensin and lysozymes). (6) They possess antioxidant properties, promoting the synthesis of protection mechanisms against reactive oxygen species (ROS), glutathione S-tranferase (GSTs), NAD (P) H: quinone reductase (NQO1) and glutamicylcysteine ligase gamma (gGCL), among others. (7) Goblet cells produce intestinal mucus. (8) L cells stimulate the synthesis of GLP-1. (9) Macrophages and dendritic cells use butyrate, stimulating the production of IL-10 and retinoic acid that also recruit lymphocytes and participate in the homeostasis of the immune system. (10) Probiotics synthesize vitamins (K, B5, B8, B9 and B12) and other substances such as lactic acid and hydrogen peroxide that act as antimicrobials. (11) They can compete and fight against pathogens. *IL-6: Interleukin-6; TNF-α: Tumor Necrosis Factor alpha, IFN-γ: Interferon gamma; TGFB: Transforming growth factor beta; IL18: Interleukin-18.

In 1965, Lilly and Stillwell were pioneers in terms of the notion of “probiotics”, referring to “secreted substances by a microorganism thar stimulate the growth of other microorganisms” [17]. Nowadays, The World Health Organization defines probiotics as “live microorganisms that, when administered in adequate amounts, confer a health benefit on the host” [18]. The microorganisms in probiotics are among the main components of fermented foods. These have been shown to have multiple effects on host gut mucosa, e.g., increasing mucus production and enhancing barrier integrity, as well as modulating the immune system by regulating cytokine production, as shown in Figure 1 [19]. Numerous studies performed with microorganisms with putative probiotic properties, such as *Lactobacillus*, *Bifidobacterium*, *Saccharomyces*, *Enterococcus*, *Streptococcus*, *Pediococcus*, *Leuconostoc*, *Bacillus* and *Escherichia coli*, have demonstrated that microorganisms can reach the gastrointestinal tract [20,21,22,23]. The potential benefits in the gut are as follows: improving the conditions and maintenance of homeostasis of the gastrointestinal tract; antagonizing activity against pathogenic species; and decreasing adherence of pathogens and antioxidant activity [20,24,25,26]. According to Dimidi et al. [2], probiotics exert a physiological benefit in the gut through competition with pathogenic bacteria and the products of immune-regulatory and neurogenic fermentation [2]. Therefore, the probiotic potential of these foods should be considered, especially for medicinal and preventive purposes. Indeed, the risk of gastrointestinal diseases can be diminished by the creation and maintenance of intestinal homeostasis of the host. Finally, probiotics are not only the main components in TFFB; prebiotics are also present.

**Figure 2:**
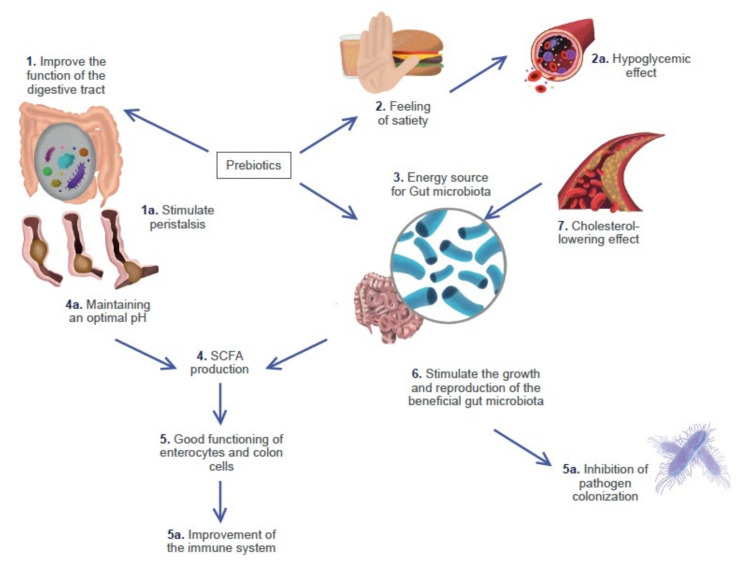
Functions of prebiotics. (1) The regulation of intestinal transit by water retention, which improves stool consistency and makes movements more fluid (1a), thereby stimulating peristalsis. (2) In adequate amounts, a feeling of satiety is given. They inhibit the absorption of simple carbohydrates and reduce blood glucose. (3) With a good source of energy, the gut microbiota achieves a proper function. (4) The production of SCFA (4a) has an impact on the intestinal pH (which, under optimal conditions, is slightly acidic), leading to the inhibition of the proliferation of pathogens. (5) SCFAs are a source of energy for enterocytes and colonocytes, (5a) improving the immune system. (6) They stimulate the growth and reproduction of beneficial gut microbiota, (6a) inhibiting the colonization of pathogenic bacteria. (7) The proper function of the gut microbiota induces hypocholesterolemia.

Prebiotics were first described by Gibson and Roberfroid in 1955 as a “non-digestible food ingredient that beneficially affects the host by selectively stimulating the growth and/or activity of one or a limited number of bacteria in the colon, and thus improves host health” [27]. Recently, prebiotics have been defined as non-digestible and non-hydrolysable carbohydrates such as galacto-oligosaccharides, fructo-oligosaccharides, soybean oligosaccharides, inulin, ciclodextrins, gluco-oligosaccharides, xylo-oligosaccharides, lactulose, lacto-sucrose, isomaltooligosaccharides, fructans and arabinoxylan; these functions are demonstrated in Figure 2. Prebiotics help to reduce constipation, foster weight gain or loss, improve glucose and lipids control, stimulate the immune system and increase the absorbability of calcium. Additionally, it has been reported that probiotics have an anticarcinogenic effect [19]. Some of the favorable effects of prebiotics, when used to colonize the host, are their ability to generate metabolites, such as short-chain fatty acids (SCFA), i.e., carbon sources in the colon which play diverse biological roles [28]. The components of prebiotics, i.e., polyunsaturated fatty acids (PUFAs), may influence diverse aspects of immunity and metabolism [29].

Several experimental studies that have used TFFB have reported associations with benefits on human health. Those studies revealed a significant link between the consumption of fermented foods and weight maintenance [30,31]. Likewise, other studies have shown reductions in the risk of cardiovascular disease [32,33,34], type 2 diabetes (T2D) [11,35,36,37], cancer [16,29] and most importantly, mortality [38,39,40].

Therefore, the aim of this review is to analyze existing information from experimental studies that comprised interventions using various TFFB and the association thereof with human health.

## 2. Microorganisms Found in Traditional Fermented Foods and Beverages

It is important to note that microorganisms are responsible for the characteristics of fermented foods and beverages. In other words, microorganisms delimit acidity, flavor and texture. Nowadays, the most important roles of TFFB are their health benefits that go beyond simple nutrition [41]. Table 1 presents an overview of various TFFB, including a description, their identified microorganisms and their region of origin.

### 2.1. Africa

Africa is one of the continents that depends most on fermented food and other conservation methods to achieve a satisfactory diet. “Ogi”, “iru” and “gari” are fermented Nigerian foods that have been widely commercialized. However, many others are made at the household level. Ogi is a fermented corn, sorghum or millet grains. Iru is a fermented product of African carob, and gari is a fermented cassava product derived from peeled fresh roots grated to a puree that is place inside of bags for fermentation. The main microorganisms in these products are lactic acid bacteria (LAB) [43] and yeasts [43,44,72,79].

“Borde” and “Shamita” are important traditional fermented Ethiopian drinks, produced by the overnight fermentation of certain cereals by LAB [43,80].

“Togwa” is a fermented beverage from Tanzania. It can be prepared based on cassava, corn, sorghum and millet, or combinations of these. Yeasts and lactic acid bacteria are the predominant microorganisms found in Togwa [43,81]. “Amasi” or sour milk, “umqombothi” or sorghum beer, and “andamahewu”, a non-alcoholic fermented cornmeal, are South African foods and drinks. “Amasi” is produced by using specific *Lactobacillus* such as *Lactobacillus delbrueckii* subsp. *lactis* and *Streptococcus* spp. “Amahewu” is made with an initial culture of LAB, while “umqombothi” is made from corn or sorghum by fermentation with wild yeast and LAB from malted sorghum adjuncts [79,80,82].

Sour porridge is a corn or sorghum food which is fermented using mainly LAB to improve and develop the palatability, flavor and nutrition. “Chibuku” is a traditional Zimbabwe sorghum beer commercially produced by fermentation with sorghum yeast, while “mabisi”, “munkoyo” and “chibwantu” are traditional Zambian foods and beverages produced through fermentation with yeast and lactic acid [79].

### 2.2. America

A large number and variety of TFFB are produced in the Americas, many of which come from pre-Hispanic times. Therefore, they are deeply rooted in the customs of most Latin Americans. An example of this is the “atole agrio”, a drink consumed in Central America. Atole agrio is a fermented beverage of corn in water seasoned with aromatic spices and other flavorings (chocolate, juice, or sweet fruit pulp). It is also the base for other pre-Hispanic drinks and forms one of the most typical breakfasts in Latin America [56,64].

“Chicha” is consumed throughout Latin America, most frequently in northern Argentina. It is obtained from the fermentation of corn. Another important South American drink is “masato”, which is consumed mainly in Colombia, Peru and Venezuela; it is made from cassava, rice, corn or pineapple [56,61,64].

Mexico is one of the countries with the largest number of TFFB; these include “tepache” (pineapple), “pozol, tesguiño and atole agrio” (corn), “pulque” (Agave), and “colonche” (red prickly), among others [8,56]. The microorganisms found in these TFFB might be different, even if they come from the same geographical region, due to their artisanal preparation. The microorganisms present in these TFFB are *Lactobacillus*, *Bifidobacterium*, *Bacillus* and yeasts [61].

### 2.3. Asia

In Asia the most common microorganisms found in fermented food are *Lactiplantibacillus plantarum*, *Levilactobacillus brevis*, *Pediococcus cerevisiae*, *Acetobacter* and *Enterobacter*. Especially in Korea, China and Nepal, these microorganisms are found in fermented vegetables. In Indonesia and Japan, these microorganisms can be found in fermented soybeans and rice wine, and in China in fermented tea, as described in Table 1 [3].

### 2.4. Europe

European countries have a variety of products containing prebiotics or probiotics, such as fermented olives, milk, cheese, meat and bread, among others. In all of these, *Lacticaseibacillus paracasei* is the most predominant microorganism. Nowadays, kefir is one of the most popular products containing microorganisms from the Lactobacillaceae family, such as *Lentilactobacillus kefiri*, *Lacticaseibacillus paracasei*, *Lentilactobacillus* *parabuchneri*, *Lacticaseibacillus casei*, *Lactobacillus lactis*, *Lactococcus lactis*, *Acetobacter lovaniensis*, *Kluyveromyces Lactis* and *Saccharomyces cerevisiae* [83].

As mentioned, TFFB make a major contribution to dietary staples in numerous countries around the world. Fermented foods and beverages contain components that improve their nutritional qualities by modulating specific functions in the body.

## 3. Interventions Involving the Use of Traditional Fermented Foods and Beverages and Their Association with Benefits on Human Health

The use of TFFB to promote health benefits has become more widespread. Evidence supports their positive effects on human health [82,83,84,85,86]. The results of recent studies showed that fermented foods and beverages impact the health of consumers due to the presence of prebiotics and probiotics [19,24,25,26,32,34,36,37]. Several experimental studies, carried out in both humans and animals, have shown that TFFB have beneficial effects in terms of modulating immune response and metabolic function. Additionally, they can improve body fat and mass, as well as blood pressure indices and brain health, providing, for example, stress relief and memory enhancement, as well as reduced risk of anxiety, depression, behavioral dysfunctions and cancer [83,85,86,87,88]. The mechanism of action involves decreasing the production of anti-inflammatory cytokines, which play an important role in the prevention of these diseases. Also, probiotics and prebiotics have a unique metabolic capacity, i.e., they have the ability to colonize, grow and stimulate the activity of the gastrointestinal tract and immune system modulation [20,40,79].

New discoveries and advances strongly suggest that gut microbiota play an active role not only in adiposity, but also in glucose and lipid metabolism and the maintenance of the immune system and cardiovascular system, among others [89]. We will now address related studies, sorted by their continent of origin:

### 3.1. Related Studies in America

Due to promising outcomes obtained in experimental studies, potential treatments of chronic diseases using functional food have been proposed. In a study done by Padilla-Camberos et al. [30], it was reported that the ingestion of *Agave* fructans decreased the body mass index (BMI), i.e., decreased total body fat and triglycerides levels in adults with obesity (*n* = 28) who consumed a low-calorie diet (*p* < 0.001) [30]. Reimer et al. [90] also used inulin-type fructans (ITF) and whey protein in isocaloric snack bars. In their study of adults with overweight and obesity (*n* = 125), although no significant change in energy intake, body weight or BMI were observed (in contrast to the previous work), they did observe significant improvements in some aspects of appetite control following the addition of ITF and/or whey protein into snack bars (*p* < 0.02), as well as changes in gut bacterial composition and function [90].

In a study by Márquez-Aguirre et al. [89], in a group of *n* = 60 mice with high-fat diet-induced obesity, using fructans from *Agave tequilana*, it was proved that higher and lower intake of *Agave* fructans had complementary effects on metabolic disorders related to obesity (*p* < 0.05) [89].

In 2015, López-Velázquez et al. [91] carried out a randomized, double-blinded controlled trial in newborns to assess the prebiotic activity of *Agave tequilana* (Metlin and Metlos Mexican product ^®^) for three months. The sample comprised 600 newborns, divided into the following groups: group 1, formula-fed with added probiotics (*Lacticaseibacillus rhamnosus*) + Metlin + Metlos; group 2, formula-fed with added probiotics + Metlin; group 3, formula-fed with added probiotics + Metlos; group 4, formula-fed with added probiotics; group 5, formula-fed without probiotics or prebiotics. A sixth group was also included as a positive control for breastfeeding. The results demonstrated statistically significant (*p* < 0.005) beneficial differences, especially in group 1, including a direct impact on the immune response (salivary IgA), bone metabolism, lower levels of total cholesterol, triglycerides and lipoproteins [91].

Martínez-Abundis et al. [92] conducted a study in adults with dyslipidemia. The intervention design consisted of two groups treated for 12 weeks: Group 1, simavastatin + *Agave* inulin + ezetimibe placebo; and Group 2 Simavastatin + Ezetimibe + *Agave* inulin placebo. The result showed a significant decrease in total cholesterol in both groups (group 1: 235 ± 29 vs. 182 ± 42 mg/dL; (*p* = 0.001) and group 2: 236 ± 31 vs. 160 ± 48 mg/dL; (*p* < 0.001)). Regarding low-density lipoprotein cholesterol, the following results were recorded: in Group 1, 141 ± 32 vs. 99 ± 34 mg/dL (*p* < 0.001); and in Group 2, 149 ± 35 vs. 89 ± 43 mg/dL (*p* <0.001). For triglycerides, the results were as follows: Group 1, 284 ± 117 vs. 214 ± 137 mg/dL (*p* = 0.027); and Group 2, 241 ± 81 vs. 180 ± 68 mg/dL; (*p* < 0.001) [92]. In a study by Contreras-Haro et al. [93] in 14 patients with obesity, changes in postprandial ghrelin levels were observed after inulin consumption from *Agave tequilana* [93].

Beltrán-Barrientos et al. [94] carried out a study with fermented milk in prehypertensive subjects. Participants were randomized into two groups (*n* = 18 each group): one group was treated with milk fermented with *Lactococcus lactis* NRRL B-50571, while a control group was treated with artificially acidified milk for 5 weeks. The results showed that the systolic ((116.5 ± 12.26 mmHg vs. 124.77 ± 11.04 mmHg) and diastolic (80.7 ± 9 vs. 84.5 ± 8.5 mmHg)) blood pressure of the group treated with fermented milk was lower than that of the control group. Additionally, other parameters such as triglycerides, total cholesterol and low-density lipoproteins in the blood serum were lower in the group treated with fermented milk compared to the control group [94]. This effect was also described by Rodríguez-Figueroa et al. [95] in rats, where it was observed that rats presented significantly reduced blood pressure after 4 weeks of ingestion of milk fermented with *Lactococcus lactis* NRRL B-50572 [95].

### 3.2. Related Studies in Asia

TFFB are particularly popular in Asia. On this continent, techniques have been developed for preserving cereals, vegetables, and meat. TFFB provide benefits to human health such as microbial stability, nutritional content and detoxification, among others [68]. *Aspergillus*, *Rhizopus*, *Mucor*, *Amylomyces*, and Bacilos are the principal microorganisms found in fermented vegetables, milk, cereal products, soybean food, starters and alcoholic beverages, as shown in Table 1 [68,96].

In a study by Rahat-Rozenbloom et al. [96], 25 male and non-pregnant, non-lactating females with BMI ≥ 20 and ≤35 kg/m^2^ were divided in two groups: 12 participants in the lean group and 13 in the obese group. The participants were studied for 6 h on three separate days after consuming 300 mL water containing 75 g glucose (GLU) as a control or with 24 g inulin (IN) or 28 g resistant starch (RS). In the study, it was found that RS had favorable second-meal effects which were likely related to changes in free fatty acids rather than short-chain fatty acid concentrations (*p* < 0.001) [96]. Yang et al. [31] also used inulin and *Camellia sinensis* with a group of men and women (*n* = 30) and found that the continuous intake of catechin-rich green tea in combination with inulin for at least 3 weeks may be beneficial for weight management (*p* < 0.05) [31].

Regarding kombucha, experimental evidence suggests different properties such as antioxidant, energizing potencies, and promotion of depressed immunity [97]. Nevertheless, it is important to emphasize that this evidence was obtained via animal and in vitro experiments, not in humans [67,72,97,98]. On the other hand, Chungkookjang showed improvements in visceral fat, lean body mass and percentage of body fat in human patients with obesity [73,99].

Kimchi, another traditional Korean fermented food, has been shown to improve insulin resistance, blood pressure, and body fat, among others. In a study performed by Han et al. [69], 24 obese women were randomly assigned to either a fresh or fermented kimchi group (80 g of fresh or fermented kimchi per day (60 g/pkg × 3 meals) for eight weeks). To verify the correlation between the anti-obesity effects of kimchi and changes in gut microbiota, fecal and blood samples were analyzed. Additionally, fecal microbiota were pyro-sequenced and microarray analyses of blood samples were done. In the study, it was found that fresh and fermented kimchi exerted differential effects on obesity-related clinical parameters. Correlations of these effects with changes in blood gene expressions and gut microbial population were more evident in the fermented kimchi group than the fresh kimchi group [69]. In addition, Kim and Park conducted a study with standardized and functional kimchi intake in adults. They observed a significant decrease in levels of LDL-C (Low-density lipoprotein-cholesterol) (*p* < 0.05) and increased levels of HDL-C (High-density lipoprotein-cholesterol) (*p* < 0.01). However, fresh kimchi intake was associated with a reduction of total serum cholesterol, triglycerides and IL-6 levels, as well as an increase in adiponectin levels (*p* < 0.05). In the fecal analysis, the standardized kimchi and functional kimchi groups showed decreased pH, β-glucosidase and β-glucuronidase levels (*p* < 0.01). Furthermore, intake of kimchi, especially functional kimchi, reduced the abundance of *Firmicutes*, but increased levels of *Bacteroidetes*. In addition, intake of both types of kimchi increased the abundance of SCFA production-related genera (*Faecalibacterium*, *Roseburia*, and *Phascolactobacterium*) and reduced *Clostridium* sp. and *Escherichia coli* group counts. Thus, the consumption of kimchi regulates metabolic parameters and colon health [70].

Tempeh, a traditional fermented soybean product, is consumed in Indonesia. It has been shown to have beneficial effects on the immune system. In a study performed in 16 participants by Tjasa Subandi et al. [100], it was reported that tempeh increased secretory immunoglobulin A (IgA) production in the ileum and colon. Tempeh acted as a potential modulator of the composition of gut microbiota, since its consumption increased the population of *A. muciniphila* in the human intestinal tracts [100].

In another study from Indonesia, a symbiotic fermented milk (skimmed milk, fructooligosaccharides (FOS) *Lactiplantibacillus plantarum*) fortified with iron and zinc was prepared to assess its effect on infant growth. The sample consisted of 94 children under five years of age with growth retardation, randomly assigned into two groups receiving their respective treatments for 3 months: intervention group (double fortified symbiotic milk) and control group (unfortified milk). It was observed that the Z score of weight/height for age in normal children of both groups increased, although the difference between groups was not statistically significant (*p* > 0.005) [101].

### 3.3. Related Studies in Africa

Africa is a continent rich in traditions and culture, as well as traditional foods. A wide range of fermented foods are produced across the continent and consumed on a daily basis [79]. Currently, there are not many clinical trials describing the health benefits of traditional African fermented foods. However, some articles have discussed the possible beneficial effects on humans of consuming these foods, based on the microorganisms that they might contain.

Ogi, iru, togwa and gari mainly contain *Lactobacillus* and yeasts. Amasi presents mostly *Lactobacillus* and *Streptococcus*. Borde, amahewu, chibuku and samita notably contain *Lactobacillus* [41,79]. Also, in most of these foods there are significant amounts of *Bifidobacterium*, *Enterococcus*, *Lactococcus* and *Leuconostoc*.

The effects of the *Lactobacillus* and *Bifidobacterium* which are present in traditional African foods include: reduction of total cholesterol and LDL, increased levels of HDL, decreased risk of dental caries, and antimicrobial and antifungal actions, in addition to the inhibition of the growth of pathogens, modulation of the immune system and mental health, reduction of mycotoxins in fermented maize products. In another study, Mokoena et al. [43], explained that these bacteria can also help as drug-delivery vehicles [43]. Potential health benefits could be found in traditional fermented foods. However, more research is needed.

### 3.4. Related Studies in Europe

Food and beverage fermentation stands as a remarkable benchmark in the history of human society. Ancient cultures such as those of Egypt, Rome, Greece and Mesopotamia used TFFB as a medicine to treat diseases [75]. Baschali et al. [75] described that “Low-Alcoholic Fermented Beverages (LAFB) and Non-Alcoholic Fermented Beverages (NAFB) are treasured as major dietary constituents in numerous European countries”. The resulting prolonged shelf-life also contributes to food security [75].

A clinical trial by Amoutzopoulos et al. [102] in Turkey showed that “hardaliye”, a traditional fermented drink based on grapes, has antioxidant effects. In the study, 100 adults between 20 and 60 years of age were randomly assigned to the following groups: High Hardaliye (HH), Low Hardaliye (LH) and control group. The HH and LH groups were composed of 45 and 35 participants who consumed a daily dose of 500 mL and 250 mL hardaliye during the study period, respectively. Twenty subjects in the control group did not receive any hardaliye. It was noted in the HH and LH groups that the measurements of conjugated dienes, malondialdehyde, and homocysteine decreased significantly (*p* = 0.001). Furthermore, they also found a significant homocysteine reduction between HH and LH. Finally, total antioxidant capacity and vitamin C increased in both groups [102].

Dönmez et al. [103] conducted a clinical trial in Turkey with “koumiss”, a traditional milk beverage produced from the fermentation of mares’ milk. Eighteen sedentary men were assigned to three equal groups: koumiss (K), exercise + koumiss (KE), and exercise alone (E). At the end of the study, triglyceride and cholesterol levels were found to have decreased in all groups, but the decrease was significant on day 15 only for the KE group. HDL cholesterol tended to increase in all groups on day 15, but the increase was significant only in the KE group (*p* = 0.001) [103].

On the other hand, a study carried out in Italy and Spain evaluated the effect of a partly fermented infant formula using bacterial strains *Bifidobacterium breve* C50 and *Streptococcus thermophilus* 065 with a specific prebiotic mixture (short-chain galacto-oligosaccharides (scGOS)) and long-chain fructo-oligosaccharides (lcFOS; 9:1). The formula was given to 200 infants ≤28 days of age; the infants were assigned either to experimental infant formula containing 30% fermented formula and 0.8 g/100 mL scGOS/lcFOS or to non-fermented control infant formula without scGOS/lcFOS groups, with infant breastfed serving as the control group. In this study, no relevant differences were found in gastrointestinal symptoms; however, stool consistency was softer in the experimental versus the control group. Daily weight gain was equivalent for both formula groups (0.5 SD margins) with growth outcomes close to those of breastfed infants. No clinically relevant differences in adverse events were observed, apart from a lower investigator-reported prevalence of infantile colic in the experimental versus the control group (1.1% vs. 8.7%; *p* < 0.02). In conclusion, they found that the partly fermented formula with prebiotics induced stool consistencies closer to those of breast-fed infants [104].

In Slovenia and Croatia, a study was done to investigate the influence of a symbiotic fermented milk on the fecal microbiota composition of 30 adults with irritable bowel syndrome (IBS). The symbiotic product contained *Lactobacilllus acidophilus* La-5, *Bifidobacterium animalis* ssp. *lacti*s BB-12, *Streptococcus thermophilus* and dietary fiber (90% inulin, 10% oligofructose), as well as a heat-treated fermented milk without probiotic bacteria, while dietary fiber alone served as placebo. Stool samples were collected after a run-in period of a 4 weeks, and at a 1-week follow-up period. After 4 weeks of symbiotic (11 subjects) or placebo (19 subjects) consumption, a greater increase in DNA specific for *Lactobacillus acidophilus* La-5 and *Bifidobacterium animalis* ssp. *lactis* was detected in the feces of the symbiotic group compared with the placebo group. At the end of consumption period, the feces of all subjects assigned to the symbiotic group contained viable bacteria with a BB-12-like RAPD profile, and after one week of follow-up, BB-12-like bacteria remained in the feces of 87.5% of these subjects. Next-generation sequencing of 16S rDNA amplicons revealed that only the percentage of sequences assigned to *S. thermophilus* was temporarily increased in both groups, whereas the global profile of the fecal microbiota of patients was not altered by the consumption of the symbiotic or placebo [105].

These studies have shown, through scientific evidence, the positive effects of TFFB on human health benefits. Moreover, the benefits of probiotics and prebiotics are promising for clinical use against noncommunicable diseases (NCDs).

## 4. Conclusions

In recent years, the intake of TFFB has revealed benefits to human health and favorable functions on NCDs, gastrointestinal, and immune disorders, suggesting that TFFB could be used to improve human diets.

Moreover, the gut microbiota composition plays an important role in metabolic disorders. Dysbiosis, or an imbalance of microorganisms in the gut microbiota associated with metabolic disorders, can potentially be modulated by probiotics or prebiotics. Several studies have shown the therapeutic effects of prebiotics and probiotics on BMI, waist circumference, accumulation of body fat, glucose and lipid levels.

TFFB are beneficial and can be used as a novel tool in the multicomponent treatment of different chronic non-transmissible diseases. However, the dosage, duration of treatment and short-long-term effects of the administration of the different microorganisms, are still a matter of research. When consumed in adequate amounts, TFFB show health benefits associated with cardiovascular diseases, type 2 diabetes, obesity and neurological problems, among others.

In conclusion, further research is needed to gain insights into the mechanisms involved in the treatment of diseases with prebiotics and probiotics. A better understanding of the relationship between the functions and the impact of TFFB is needed in order to develop strategies for the management of chronic gastrointestinal diseases, among other conditions.

## Figures and Tables

**Table 1:** Microorganisms found in common fermented foods.

Name of the Product	Description	Microorganisms Identified	Region of Origin	References
Africa				
Ogi	Fermented cereal pudding (maize, sorghum, or millet)	*Candida krusei*, *Lactiplantibacillus plantarum*, *Limosilactobacillus fermentum*, *Saccharomyces cerevisiae*, *Acetobacter* spp., *Corynebacterium* spp.	West Africa	[42]
Iru	Fermented locust beans (*Parkia biglobosa*)	*Bacillus*, *Staphylococcus* spp.	West Africa	[43]
Gari	Cassava	*Leuconostoc mesenteroide*, *Lactiplantibacillus plantarum*, *Bacillus subtilis*, *Candida krusei*	West Africa	[43]
Togwa	Cereal-based, starch-saccharified, non-alcoholic, lactic acid-containing gruel food (Cassava, maize, sorghum, millet)	*Lactobacillus* spp., *Pediococcus pentosaceus*, *Weissella confusa*, *Issatchenkia orientalis*, *Saccharomyces cerevisiae*, *Candida pelliculosa and Candida tropicalis.*	Eastern Africa	[44]
Bushera	Fermented cereal beverage (sorgum)	*Lactobacillus* spp., *Streptococcus* spp., *Leuconostoc* spp., *Pediococcus* spp., *Weissella* spp.	Eastern Africa	[43]
Shamita	Fermented beverage (barley)	*Lactobacillus* spp.	North East Africa	[45]
Munkoyo	Mildly fermented drink made from pounded roots mixed with bits of maize	*Weisella* and *Lactobacillus* spp.	Southern Africa	[46]
Mahewu	Fermented maize or sorghum beverage	*Lactiplantibacillus plantarum*, *Limosilactobacillus fermentum*	Southern Africa	[47]
Mabisi/Amasi	Fermented milk drink	*Lactococcus*, *Lactobacillus and Streptococcus* spp., *Leuconostoc* spp.	Southern Africa	[48]
Amabere amaruranu	Fermented milk drink	*Streptococcus thermophilus*, *Lactiplantibacillus plantarum*, *Leuconostoc mesenteroides*; Yeasts	Southern Africa	[49]
Marula/buganu	Fermented juice and pulp (marula fruit)	Yeasts, *Lactobacillus* spp.	Southern Africa	[50]
Motoho	Fermented sorghum beverage	*Lactiplantibacillus plantarum*, *Limosilactobacillus fermentum*, *Lactobacillus coryniformis*, *Lacticaseibacillus paracasei paracasei*, *Candida lambica*, *Candida kefyr*, *Candida glabarata*, *Candida pelliculosa; Rhodotorula mucilaginosa*, *Geotrichum*, *candidum*, *Geotrichum silvicola*	South Africa	[51]
Chibuku	Sorghum beer	*Lactobacillus* spp., *Saccharomyces cerevisiae*	Southern Africa	[52]
Umqombothi	Beer made from maize and sorghum	*Lactobacillus* spp., *Saccharomyces cerevisiae*	Southern Africa	[53]
Burukutu	Alcoholic beverage, brewed from the grains of Guinea corn (*Sorghum bicolor*) and millet (*Pennisetum glaucum*).	*Escherichia*, *Staphylococcus*, *Bacillus*, *Lactobacillus*, *Leuconostoc*, *Acetobacter*	Nigeria, Benin, Ghana	[54]
Dégué	Fermented millet dough	*Escherichia*, *Bacillus*, *Lactobacillus*, *Enterococcus*	Burkina Faso	[55]
America				
Champuz	Fermented maize or rice beverage	Unknow	Colombia, Peru	[56]
Chicha	Fermented maize beverage	*Enterococcus*, *Lactococcus*, *Streptococcus*, *Weissella*, *Leuconostoc*, *Lactobacillus*, *Saccharomyces.*	Colombia, Peru, Argentina	[57]
Kefir grain	Fermented lactoalcoholic milk	*Leuconostoc mesenteroides*, *Lactococcus lactis sp. cremoris*, *Lacticaseibacillus paracasei*	Brazil	[58]
Axokot/Atole agrio	Fermented maize beberage	*Leuconostoc*, *Lactococcus*, *Lactobacillus*, *Pediococcus*, *Weisella*	Southeast Mexico	[59]
Tepache	Fermented alcoholic pineapple drink	*Lactiplantibacillus plantarum*, *Leuconostoc mesenteroides*, *Lactobacillus* sp., *Lactococcus lactis*, *Hanseniaspora*, *Bacillus* spp., *Torulopsis*, *Saccharomyces* and *Candida*	Mexico	[60]
Tesgüiño	Maize beer	*Lactobacillus* spp. *Candida*, *Saccharomyces*, *Hansenula*	North Western and North Mexico	[61]
Tuba	Alcoholic beverage from coconut palm	Data not available	Western Mexico	[56]
Pozol	Drink based on cocoa and maize.	*Lactococcus*, *Lactobacillus*, *Leuconostoc*, *Streptococcus*, *Enterococcus*, *Weisella*, *Saccharomyces*, *Candida*, *Aspergillus*, *Penicillum*, *Rhizopus*	Southeast Mexico	[61,62]
Colonche	Fermented red prickly beverage	*Candida valida*, *Saccharomyces cerevisiae*, *Torulopsis taboadae*, *Pichia fermentans*	North of Mexico	[63,64]
Pulque	Fermented alcoholic drink (several species of *Agave*)	*Lactobacillus*, *Leuconostoc*, *Microbacterium*, *Flavobacterium*, *Acetobacter*, *Gluconobacter*, *Zymomonas*, *Saccharomyces*	Central Mexico	[65]
Asia				
Kombucha	Fermented tea beverage	*Komagataeibacter xylinus*, *Brettanomyces bruxellensis*, *Acetobacter pasteurianus*, *Acetobacter xylinum*, *Acetobater aceti*, *Saccharomyces cerevisiae*, *Zygosaccharomyces bailii*, *Zygosaccharomyces* spp., *Gluconacetobacter*	China	[2,66,67]
Resistant starch	Insoluble type of cereal fiber from grains	*FOS*	Japan, EUA, Europe.	[68]
Catechin-rich green tea	Green tea from *Camellia sinensis plant*	Epicatechin (EC) Epicatechin-3-gallate (ECG) Epigallocatechin (EGC) Epigallocatechin-3-gallate (EGCG)	China	[31]
Kimchi	Cabbage, radish, various vegetables	*Leuconostoc mesenteroides*, *Levilactobacillus brevis*, *Lactiplantibacillus plantarum*	Korea	[69,70]
Tempeh	Fermented boiled and dehulled soybeans	*Enterococcus faecium*, *Rhizopus oryzae*, *Rhizopus oligoporus*, *Mucor indicus* *Mucor circinelloides*, *Geotrichum candidum*, *Aureobasidium pullulans*, *Alternaria alternata*, *Cladosporium oxysporum*, *Trichosporon beigelii*, *Clavispora lusitaniae*, *Candida maltosa*, *Candida intermedia*, *Yarrowia lipolytica*, *Lodderomyces elongisporus*, *Rhodotorula mucilaginosa*, *Candida sake*, *Hansenula fabiani*, *Candida tropicalis*, *Candida parapsilosis*, *Pichia membranefaciens*, *Rhodotorula rubra*, *Candida rugosa*, *Candida curvata*, *Hansenula anomola*	Indonesia	[2]
Khalpi	Cucumber	*Lactiplantibacillus plantarum*, *Levilactobacillus brevis*, *Leuconostoc fallax*	Nepal	[71,72]
Chungkookjang	Fermented soybean	*Bacillus subtilis*, *Bacillus licheniformis*	Korea	[73]
Miso	Fermented soybean paste	*Bacillus subtilis*, *Bacillus amyloliquefaciens*, *Staphylococcus gallinarum*, *Staphylococcus kloosii*, *Lactococcus* sp. *GM005*	Japan	[2]
Sake	Rice wine	*Fructilactobacillus fructivorans*, *Lactobacillus homohiochi.*	Japan	[66]
Burong mustala	Mustard leaf	*Levilactobacillus brevis *	Philippines	[71]
Europe				
Sourdough bread	Bread made from longer ferment	Data not available	Middle East and Europe	[2,74]
Fermented olives	Olives	*Lactiplantibacillus plantarum*, *Lactobacillus pentosus*, *Lacticaseibacillus casei*	Spain and Portugal	[74,75]
Salsiccia, Soppressata	Chopped pork	*Micrococci*, *Staphylococci*	Italy	[74]
Kefir	Fermented milk beverage	*Lentilactobacillus kefiri*, *Lacticaseibacillus paracasei*, *Lactobacillus parabuchneri*, *Lacticaseibacillus casei*, *Lactobacillus lactis*, *Acetobacter lovaniensis*, *Kluyveromyces lactis*, *Saccharomyces cerevisiae*	Russia, Europe, Middle East	[76]
Fermented Cheese	Milk	*Lactococcus lactis*, *Bifidobacterium bifidum*, *Lactobacillus acidophilus*, *Lacticaseibacillus paracasei*	Europe, Middle East	[77,78]
Sauerkraut	Fermented cabbage	*Lactiplantibacillus plantarum*	Germany	[6]

## Data Availability

Not applicable.
